# Near 100% CO selectivity in nanoscaled iron-based oxygen carriers for chemical looping methane partial oxidation

**DOI:** 10.1038/s41467-019-13560-0

**Published:** 2019-12-03

**Authors:** Yan Liu, Lang Qin, Zhuo Cheng, Josh W. Goetze, Fanhe Kong, Jonathan A. Fan, Liang-Shih Fan

**Affiliations:** 1 Department of Chemical and Biomolecular Engineering, The Ohio State University, 151W Woodruff Ave, Columbus, OH 43210 USA; 2 Department of Electrical Engineering, Ginzton Laboratory, Spilker Engineering and Applied Sciences, Stanford University, 348 Via Pueblo Mall, Stanford, CA 94305 USA

**Keywords:** Heterogeneous catalysis, Devices for energy harvesting, Chemical engineering, Porous materials

## Abstract

Chemical looping methane partial oxidation provides an energy and cost effective route for methane utilization. However, there is considerable CO_2_ co-production in current chemical looping systems, rendering a decreased productivity in value-added fuels or chemicals. In this work, we demonstrate that the co-production of CO_2_ can be dramatically suppressed in methane partial oxidation reactions using iron oxide nanoparticles embedded in mesoporous silica matrix. We experimentally obtain near 100% CO selectivity in a cyclic redox system at 750–935 °C, which is a significantly lower temperature range than in conventional oxygen carrier systems. Density functional theory calculations elucidate the origins for such selectivity and show that low-coordinated lattice oxygen atoms on the surface of nanoparticles significantly promote Fe–O bond cleavage and CO formation. We envision that embedded nanostructured oxygen carriers have the potential to serve as a general materials platform for redox reactions with nanomaterials at high temperatures.

## Abstract (web summary)

Chemical looping methane partial oxidation is an effective technology to produce syngas with a minimal energy penalty. Here, the authors design and develop a mesoporous silica supported nanoparticle oxygen carrier that enables a near 100% CO generation with high recyclability and substantially lower operating temperature.

## Introduction

Syngas, i.e., CO and H_2_, is an important intermediate for producing fuels and value-added chemicals from methane via Fischer–Tropsch or other synthesis techniques^1^. Syngas has been produced commercially by steam reforming, autothermal reforming, and partial oxidation of methane for many decades^2^. However, an improvement of its energy consumption, environmental impact, and associated production cost has always been of interest. This has prompted the investigation into alternative routes that can avoid the use of air separation units for producing purified oxygen and are more effective in CO_2_ emission control. It is also of interest to reduce the operating temperature of these processes, which are generally endothermic and traditionally require temperatures of 900 °C or higher to attain high reactant conversion rates. The use of high temperatures is problematic because the thermodynamic driving force for carbon deposition, and thus materials obliteration, can be accelerated^3^. Current approaches to reducing reaction temperatures while avoiding side product formation require noble metals such as Pt, Pd, or Au, which leads to dramatic increases in cost^4^.

**Fig. 1:**
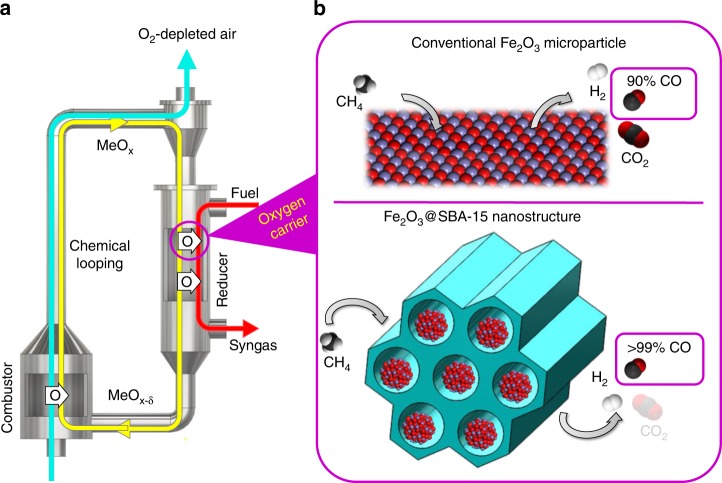
Chemical looping partial oxidation with methane. **a** Schematic of the chemical looping partial oxidation process; **b** structure and CO selectivity in conventional oxygen carrier vs Fe_2_O_3_@SBA-15 oxygen carrier.

Chemical looping methane partial oxidation^5^ (CLPO) is an emerging approach that overcomes the above-mentioned shortcomings for syngas production. A CLPO process involves redox reactions taking place in two interconnected reactors: a reducer (or fuel reactor) and an oxidizer (also referred to as air reactor), shown in Fig. 1a. In contrast to conventional fossil fuel gasification and reforming processes, CLPO eliminates the need for an air separation unit, water–gas shift reactor, and acid gas removal unit. It has the potential to directly produce high-quality syngas with desirable H_2_:CO ratios. The core of CLPO using natural gas as the feedstock involves complex redox reactions in which methane molecules adsorb and dissociate on metal oxide oxygen carrier surfaces. It also involves internal lattice oxygen ion diffusion in which oxygen vacancy creation and annihilation occurs. These reactions can be engineered to withstand thousands of redox cycles^5^.

The recent progress in chemical looping technology for partial oxidation has advanced to the stage where successful pilot operation has been demonstrated and proven to be highly efficient with a minimal energy penalty in the process applications^6^. In this technology, the highest CO selectivity that the CLPO can reach is thermodynamically limited to 90% with accompanied 10% CO_2_ generation. It is recognized that the CO_2_ reduction in low purity syngas is extremely challenging and energy consuming, as CO_2_ is among the most chemically stable carbon-based molecules^7^. This 10% of CO_2_ in syngas can significantly reduce the productivity of value-added fuels or chemicals generated. Breaking away from the 90% CO yield limits to achieve near 100% CO selectivity requires a different consideration from the metal oxide materials design and synthesis perspective.

We report an approach to metal oxide oxygen carrier engineering for CLPO by designing and synthesizing nanoscale iron oxide carriers^8^ embedded in mesoporous silica SBA-15 (Fe_2_O_3_@SBA-15). Mesoporous silica is an engineered nanomaterial that has high surface area, ordered pore structures, and high tunability of morphology. Its unique properties have attracted broad attention in a number of applications such as environmental treatment, catalysis, and biomedical engineering^9–17^. SBA-15 is a common mesoporous silica that has perpendicular nanochannels with a narrow pore size distribution which is suitable for nanoparticle separation and gas penetration. A schematic of our materials platform is outlined in Fig. 1a. We experimentally achieve a high CO selectivity >99%, which is by far the highest in CLPO systems (Fig. 1b). We also find that cyclic methane partial oxidation with nanoscale oxygen carrier materials can be performed with high selectivity at temperatures as low as 750 °C. These findings underscore the strong size-dependent effects of metal oxide oxygen carriers at the nanoscale on syngas selectivity and reactant conversion in redox processes. This work will have broader impacts not only on CLPO, but also on other chemical looping applications such as carbonaceous fuel conversion and utilization.

## Results

### Characteristics of Fe_2_O_3_@SBA-15

**Fig. 2:**
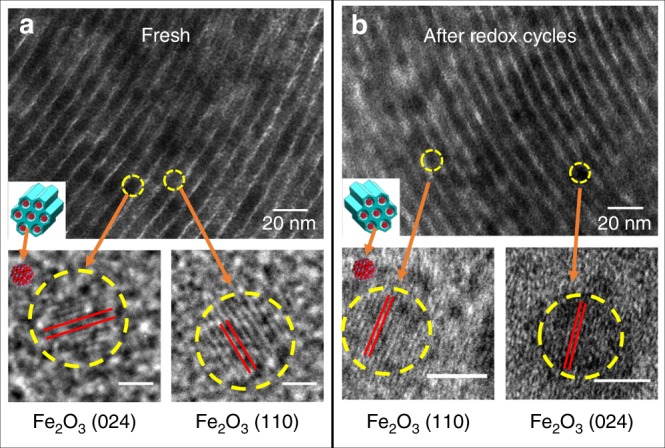
Morphological characteristics of Fe_2_O_3_@SBA-15. **a** Fresh Fe_2_O_3_@SBA-15 and HR-TEM images of two typical Fe_2_O_3_ nanoparticles (Scale bar represents 1 nm); **b** Fe_2_O_3_@SBA-15 after 75 redox cycles and HR-TEM images of two typical Fe_2_O_3_ nanoparticles (Scale bar represents 5 nm).

Figure 2 shows the transmission electron microscope (TEM) images of Fe_2_O_3_@SBA-15 before and after redox cycles with no obvious morphological distinction. Nanoparticles with a size of 3–5 nm can be identified as *α*-Fe_2_O_3_ based on lattice fringes measurement in which *a* = *b* = 5.038 Å and *c* = 13.772 Å. Element mapping is presented in Supplementary Fig. 1, suggesting that Fe_2_O_3_ nanoparticles are embedded in SBA-15 nanochannels. The TEM images also confirm that the nanoparticles remain embedded in SBA-15 nanochannels with identical morphology after 75 redox cycles. The particle size slightly increases to 5–8 nm after 75 redox cycles due to unavoidable morphology evolution. This result indicates the high stability of Fe_2_O_3_@SBA-15 at high temperatures.

The mesoporous silica SBA-15 (Supplementary Fig. 2) exhibits a surface area of 550 m^2^ g^−1^, a uniform pore size of 8 nm, and a pore volume of 0.66 cm^3^ g^−1^ (Supplementary Methods). The pore volume decreases to 0.52 cm^3^ g^−1^ which is 23% less than SBA-15 after Fe_2_O_3_ nanoparticle loading, implying a Fe_2_O_3_ nanoparticle volume loading of ~20%. The average pore size remains at 8 nm with a minor decrease in magnitude, indicating that the silica nanochannels are partially filled by Fe_2_O_3_ nanoparticles.

In comparison, Supplementary Fig. 3a, c suggests that the unsupported Fe_2_O_3_ nanoparticles are agglomerated with a wide size distribution of 50–100 nm. Supplementary Fig. 3b, d reveals that the nanoparticles will evolve to microparticles of 1–10 µm after 75 redox cycles. On the other hand, the morphology of Fe_2_O_3_@SBA-15 arrays before and after 75 redox cycles (Supplementary Fig. 3e, f) remain almost unchanged, indicating high recyclability of Fe_2_O_3_@SBA-15 at both nanoscale and microscale.

### Methane CLPO with Fe_2_O_3_@SBA-15

**Fig. 3:**
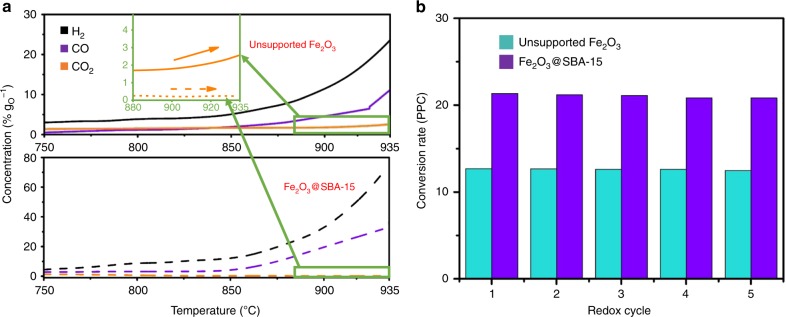
Reactivity and selectivity comparison between Fe_2_O_3_@SBA-15 and bulk Fe_2_O_3_. **a** Temperature programmed reduction results of Fe_2_O_3_@SBA-15 and unsupported Fe_2_O_3_; **b** conversion rate during redox at 800 °C.

The reactivity and recyclability test results are shown in Fig. 3. A stable CO_2_ concentration of <0.7% g_O_^−1^ is observed in Fe_2_O_3_@SBA-15 throughout methane partial oxidation, indicating a high syngas selectivity higher than 99.3%. In unsupported Fe_2_O_3_, CO_2_ formation is observed to increase with temperature, resulting in an average selectivity less than 87%. In the range of 750–935 °C, CO concentration from Fe_2_O_3_@SBA-15 is over 200% higher than unsupported Fe_2_O_3_, suggesting a near 100% CO selectivity with significantly higher CO conversion rate.

Over 75 continuous redox cycles were carried out on both Fe_2_O_3_ samples with and without SBA-15 support (Supplementary Fig. 4). Five typical TGA redox cycles are shown in Fig. 3b. The high recyclability is consistent with TEM observation. Fe_2_O_3_@SBA-15 not only has a high conversion rate, but is stable at high temperatures. This suggests that the separation of nanoparticles is essential in maintaining high CO selectivity, reactivity, and recyclability. At 800 °C, the conversion rate of Fe_2_O_3_@SBA-15 is 67% higher than unsupported Fe_2_O_3_. The morphology of post redox nanoparticles can be found in Fig. 2b, where Fe_2_O_3_@SBA-15 nanochannels remain almost identical to fresh samples. This demonstrates the high stability and anti-sintering effect of dispersed nanostructures at high temperatures. The average pore size of Fe_2_O_3_@SBA-15 is 7.6 nm after 75 redox cycles (Supplementary Fig. 5), which confirms high cyclic stability.

The methane conversion and syngas selectivity of both unsupported Fe_2_O_3_ and Fe_2_O_3_@SBA-15 were also evaluated in a quartz U-tube (1 cm diameter) fixed bed reactor. As illustrated in Supplementary Fig. 6, a high selectivity near 100% in Fe_2_O_3_@SBA-15 is confirmed in both TGA and fixed bed reactor and a higher methane conversion is also obtained compared with unsupported Fe_2_O_3_ with different weight hourly space velocity (WHSV).

### Size-dependent reaction modeling

**Fig. 4:**
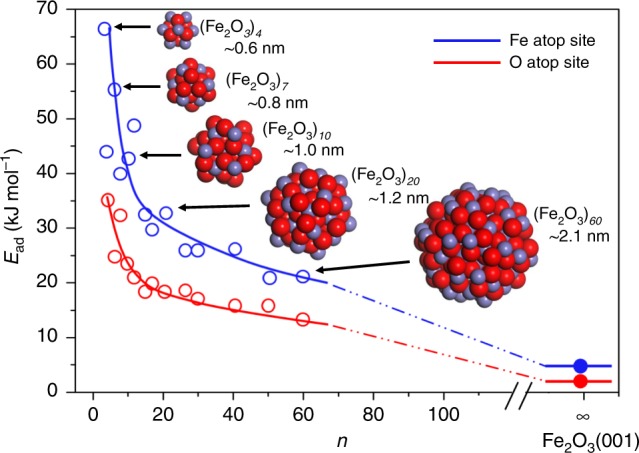
Calculated energies of CH_4_ adsorption. *E*_ad_ (kJ mol^−1^), on Fe atop site and O atop site of (Fe_2_O_3_)_*n*_ nanoparticles as a function of *n*. The adsorption trends are shown by the blue and red lines.

**Fig. 5:**
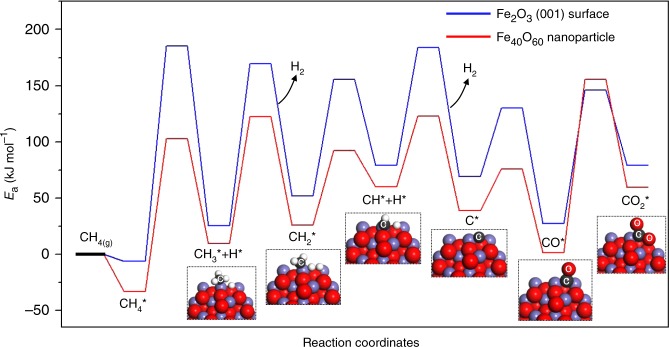
Energy profile of CH_4_ partial oxidation on Fe_40_O_60_ nanoparticle and Fe_2_O_3_ (001) surface.

To gain mechanistic insight into the role of the nanostructures in CO selectivity enhancement of Fe_2_O_3_@SBA-15, first-principles calculations were performed within the framework of density functional theory (DFT) using the Vienna Ab Initio Simulation Package (VASP)^18–20^. To take into consideration realistic experimental conditions (800 °C for CH_4_ partial oxidation), the temperature effect is included by explicitly taking into account adsorbed CH_4_ molecules in terms of ab initio atomistic thermodynamics. The theoretical description requires the size and geometry of the model to be as realistic as possible, for direct comparison with experimental samples. However, modeling (Fe_2_O_3_)_*n*_ nanoparticles of realistic size (>3 nm) by first-principles calculations is very demanding and the global optimization is hardly feasible. In this work, (Fe_2_O_3_)_60_ (~2 nm) is modeled as the largest nanoparticle to examine the size effect. Figure 4 shows calculated energies of CH_4_ adsorption on Fe atop site and O atop site of (Fe_2_O_3_)_*n*_ nanoparticles as a function of *n*, and the corresponding adsorption geometries are described in Supplementary Fig. 7. The data of the previous computational study on CH_4_ adsorption on Fe_2_O_3_ (001) surface are given by the filled circle^21^. It can be seen that CH_4_ adsorption energies dramatically decrease with increasing number, *n* when the sizes of Fe_2_O_3_ nanoparticles are at a relatively small scale. However, they decrease slowly with increasing *n* when the sizes are at relatively large scale. The strongest adsorption on (Fe_2_O_3_)_4_ is CH_4_ binding at the Fe atop site with an adsorption energy of 66.2 kJ mol^−1^. The second stable configuration is CH_4_ adsorption at the O atop site of (Fe_2_O_3_)_4_ with an adsorption energy of 35.1 kJ mol^−1^. When *n* increases from 4 to 60, the Fe atop adsorption becomes weaker with 43.9 kJ mol^−1^ lower adsorption energy. However, the adsorption at the Fe atop site and the O atop site of (Fe_2_O_3_)_60_ nanoparticles is still stronger than adsorption on Fe_2_O_3_ (001) surface, as shown in Fig. 5. This is because the average coordination number of surface Fe atoms in (Fe_2_O_3_)_*n*_ nanoparticle is smaller than that on Fe_2_O_3_ (001) surface. The undercoordination results in an upward shift of the Fe d-band, yielding high binding energies.

In chemical looping partial oxidation process, CH_4_ is initially dissociated over oxygen carriers to form hydrogen and CH_*x*_ radicals. Then the CH_*x*_ radicals are oxidized by lattice oxygen to generate CO or CO_2_^4,22^. Since the energy and geometry of CH_4_ adsorption on (Fe_2_O_3_)_20_ are similar with CH_4_ adsorption on (Fe_2_O_3_)_60_, Fe_40_O_60_ (*n* = 20) nanoparticles are chosen as the model to calculate the reaction barriers of CH_4_ dissociation and oxidation. The energy profile was mapped out as shown in Fig. 5. The energy barrier for the first step of CH_4_ dissociation on Fe_40_O_60_ is 135.8 kJ mol^−1^, which is 55.7 kJ mol^−1^ lower than that of CH_4_ dissociation on the Fe_2_O_3_ (001) surface. Fe_40_O_60_ also exhibit higher activity for CH_3_, CH_2_, and CH dissociation due to the lower barriers, compared with the Fe_2_O_3_ (001) surface. It indicates that nanostructured Fe_2_O_3_ facilitates CH_4_ conversion compared with bulk Fe_2_O_3_, which is in good agreement with TGA and fixed bed test. After methane dissociation, all C–H bonds are cleaved to generate a carbon atom and four hydrogen atoms. Fe_40_O_60_ has three chemically distinguishable types of lattice oxygen atoms: 2-fold coordinated lattice oxygen O_2C_, 3-fold coordinated lattice oxygen O_3C_, and 4-fold coordinated lattice oxygen O_sub_. As such, there are three reaction pathways for CO formation as shown in Supplementary Fig. 8. The calculated barriers for CO_2C,_ CO_3C_, and CO_sub_ formation are 37.2 kJ mol^−1^, 69.6 kJ mol^−1^, and 58.5 kJ mol^−1^, respectively. This result indicates C binding to O_2C_ is the most favorable path, compared with C binding to O_3C_ and O_sub_ because Fe–O bonds of low-coordinated lattice oxygen atoms are easier to break than high-coordinated lattice oxygen atoms. In contrast to Fe_40_O_60_, all lattice oxygen atoms in the topmost atomic layer of the Fe_2_O_3_ (001) surface are three-coordinated atoms. Thus, the carbon atom on the Fe_2_O_3_ (001) surface converts to CO only via binding to O_3C,_ leading to a relatively high barrier of 61.2 kJ mol^−1^ as shown in Fig. 5.

The formed CO may further react with surface lattice O atoms to form CO_2_^21,22^. For the Fe_40_O_60_ nanoparticle, the formation of CO_2_ needs to overcome a barrier of 148.9 kJ mol^−1^, which is 30.4 kJ mol^−1^ higher than that of CO_2_ formation on Fe_2_O_3_ (001) surface. The high barrier with respect to CO_2_ formation on Fe_40_O_60_ is attributed to the surface stress of nanoparticles, induced by surface atoms with unsaturated coordination. The surface stress leads to shorter and thus stronger Fe–O_3C_ bonds compared with Fe–O_3C_ bonds of the Fe_2_O_3_ (001) surface. The formation of CO_2_ on Fe_40_O_60_ is endothermic, with the calculated reaction energy of 58.2 kJ mol^−1^. These results indicate that the CO_2_ formation on Fe_40_O_60_ is both kinetically and thermochemically unfavorable. Therefore, Fe_40_O_60_ nanoparticles significantly promote CO formation while inhibiting CO_2_ production. Unfortunately, DFT-based calculations of large nanoparticles consisting of a few thousand atoms, required for confirming this conclusion, are intractable to compute even on the most powerful supercomputers. Nevertheless, the experimental evidence for the extraordinary CO selectivity of Fe_2_O_3_ nanoparticles with the size of 5 ± 3 nm indicates that nanostructuring makes Fe_2_O_3_ a more active oxide for CO production compared with bulk Fe_2_O_3_.

## Discussion

In summary, we demonstrate that Fe_2_O_3_@SBA-15 enables near 100% CO selectivity in chemical looping methane partial oxidation, which is so far the highest value in product selectivity observed for chemical looping systems. Moreover, the effective temperature for syngas generation is lowered to 750–935 °C, which is over 100 °C lower than current state-of-the-art processes. Nanoscaled oxygen carriers are presented to exhibit little high-temperature reactivity property deterioration and adaptability to broader temperature operating windows for chemical looping operation conditions. These are important factors that can contribute to significant energy-saving reactor designs. The theoretical model and calculations reveal that the structure of the nanoparticles play a key role in CO selectivity enhancement of Fe_2_O_3_@SBA-15. The CH_4_ adsorption energies and CO formation barriers depend not only on the nanoparticle size but also on the type of surface site exhibited by the nanoparticles. The small average coordination number of Fe atoms in the nanoparticle facilitates CH_4_ adsorption and activation due to an upward shift of the Fe d-band, while the low-coordinated O atoms greatly promote Fe–O bond cleavage and CO formation, leading to a significant increase in CO selectivity. These findings contribute to a nanoscale understanding of the underlying metal oxide redox chemistry for chemical looping processes, and provide a systematic strategy toward the design of robust oxygen carrier nanoparticles with superior activity and selectivity.

## Methods

### Sample preparation

Surfactant CTAB and Fe(NO_3_)_3_·9H_2_O were dissolved in 120 mL ethanol. 0.4 g SBA-15 was stirred in the solution for overnight at room temperature. This is followed by a powderization at 90 °C, and then calcination at 600 °C for 5 h. The prepared sample is marked as Fe_2_O_3_@SBA-15. Fe_2_O_3_ particles without SBA-15 support was also prepared by the same method. For Fe_2_O_3_@SAB-15, the volume loading was calculated by Eq. (1):

1 $$Volume\,loading = \frac{{m_{{\mathrm{Fe}}_2{\mathrm{O}}_3}/{\uprho}_{{\mathrm{Fe}}_2{\mathrm{O}}_3}}}{{m_{{\mathrm{SBA}} - 15} \times {\mathrm{pore}}\,{\mathrm{volume}}}}$$

where $m_{{\mathrm{Fe}}_2{\mathrm{O}}_3}$ is the total weight of Fe_2_O_3_, *m*_SBA-15_ is total weight of SBA-15, ${\uprho} _{{\mathrm{Fe}}_2{\mathrm{O}}_3}$ is density of Fe_2_O_3_.

### Thermogravimetric analysis (TGA)

The selectivity of Fe_2_O_3_@SBA-15 was tested in a SETARAM thermogravimetric analysis (TGA) device. A 20 mg sample was mounted in the TGA, and heated from 750 °C to 935 °C with a temperature ramp of 10 °C/min. 20 mL/min of CH_4_ balanced with 180 mL/min of He was used in the partial oxidation. The outlet gas composition was analyzed by mass spectrometry (MS). 75 redox cycles were also conducted on both samples to test their recyclability. During the methane partial oxidation, 50 mL/min of CH_4_ balanced with 100 mL/min of N_2_ and 50 mL/min of He carrier gases was used in a TGA to react with the sample for 5 min. For the regeneration, 100 mL/min of air balanced with 100 mL/min of N_2_ was used to oxidize the sample for 5 min. Between the reduction and the oxidation, 100 mL/min of N_2_ was used as the flushing gas to prevent the mixing of air and methane.

### Fixed bed experiment

The methane conversion and syngas selectivity of both unsupported Fe_2_O_3_ and Fe_2_O_3_@SBA-15 were also evaluated in a quartz U-tube (1 cm diameter) fixed bed reactor. Four different methane weight hourly space velocity (WHSV) of 17.8, 25, 30, 37.5 mL (${\mathrm{mg}}_{{\mathrm{Fe}}_2{\mathrm{O}}_3}$ h)^−1^ were applied to each sample. In the experiment, the solid were amounted in the center of the reactor that is placed in a tube furnace and heated to 800 °C. The outlet gas was analyzed by MS.

### Computational details

All plane-wave DFT calculations were performed using the projector augmented wave pseudopotentials provided in the VASP. The Perdew–Burke–Ernzerhof exchange-correlation functional was used with a plane-wave expansion cutoff of 400 eV^23^. Due to the valence electrons of Fe 3d state, the Hubbard *U* approach was used to correct self-interaction errors of α-Fe_2_O_3_^24,25^. The increase of *U* from 1 to 4 eV results in better agreement with the density of states by experimental inverse photoemission spectra^26^. However, a further increase in *U* will cause Fe 3d states to shift to unacceptably low energies. Therefore, *U* = 4 eV was chosen to describe the energy required for adding an extra d electron to the Fe atom. Geometry optimization of the nanoparticle was carried out at the Γ-point. All atoms were allowed to relax until the ionic forces are smaller than |0.01| eV Å^−1^, with a total energy threshold determining the self-consistency of the electron density of 1.0 × 10^−5^ eV atom^−1^. Dispersion interactions are modeled using the DFT-D3 method developed by Grimme et al.^27,28^. The calculated α-Fe_2_O_3_ bulk lattice parameters were *a* = *b* = 5.04 Å and *c* = 13.83 Å, in good agreement with the experimental values (*a* = *b* = 5.038 Å and *c* = 13.772 Å). The α-Fe_2_O_3_ (001) surface with Fe-O_3_-Fe- termination was chosen to model the iron oxide slab with a thickness of ~15 Å^29^. Fe_2_O_3_ nanoparticles were modeled by a three-dimensional periodic arrangement with a large cubic cell of 5 × 5 × 5 nm^3^ to minimize lateral interactions. The structures of the free nanoparticles (Fe_2_O_3_)_*n*_ (*n* = 4–60) were fully optimized without any symmetry constraints. The climbing-image nudged elastic band method^30,31^ is used to locate transition states of elementary steps and map out reaction pathways for CH_4_ dissociation and oxidation on the Fe_2_O_3_ nanoparticles_._ The effect of temperature (800 °C) was taken into account for adsorption and reaction barrier comparison.

### Characterization methods

The scanning electron microscope measurements were performed with FEI Helios Nanolab 600, with the voltage of 10 kV and the current of 0.17 mA. The TEM images were obtained with a FEI Tecnai G2 30 at 300 kV.

The surface area of the sample was analyzed by NOVA 4200e. The sample was first degassed at 300 °C for over 10 h. Then isothermal N_2_ adsorption was performed at −196 °C. The surface area of both SBA-15 and Fe_2_O_3_@SBA-15 was determined by Brunauer–Emmett–Teller method, while the pore size distribution was calculated by Brunauer–Joyner–Halenda method^32^ with adsorption branch.

Small-angle X-ray diffraction was conducted with Rigaku SmartLab equipped with Cu K-α radiant. Data were collected from 0.7° to 3° with a scanning rate of 0.1° per minute.

## Supplementary information

Supplementary Information

## Data Availability

The data that support the findings of this study are available from the corresponding author upon request.
