# Supplementary material for: Near 100% CO selectivity in nanoscaled iron-based oxygen carriers for chemical looping methane partial oxidation
Source: Nat Commun. 2019 Dec 3;10:5503. doi: 10.1038/s41467-019-13560-0 (PMC6890731; doi:10.1038/s41467-019-13560-0)
Supplement: Supplementary file 1 — Supplementary Information [file 41467_2019_13560_MOESM1_ESM.pdf]

Supplementary Information

Near 100% CO Selectivity in Nanoscaled Iron-Based Oxygen Carriers for Chemical Looping Methane Partial Oxidation

Liu et al.

Supplementary Methods

N_2 physisorption: The nanoparticle to SBA-15 ratio was altered between 0% and 80% and an ideal volume ratio of 20% nanoparticle has been selected. A higher loading volume caused surface sintering and low reactivity was observed in the lower loading volume.

Thermogravimetric analysis (TGA): The conversion rate of the oxygen carrier is calculated by Supplementary Equation 1:

$$Conversion\ rate = \frac{\Delta m}{m_{Fe_2O_3} \times 30\%} \times 100\% \quad (1)$$

where Δm is the weight change during redox cycle, $m_{Fe_2O_3}$ is the weight of Fe_2O_3 in the total sample, and 30% is the weight percentage of oxygen in Fe_2O_3. The unit is PPC, which stands for "percentage per cycle". The conversion rate of 75 redox cycles is shown in Supplementary Figure 4. The selectivity is calculated by Supplementary Equation 2:

$$Selectivity = \frac{x_{CO}}{x_{CO} + x_{CO_2}} \times 100\% \quad (2)$$

where x_{CO} is the mole fraction of CO product, x_{CO_2} is the mole fraction of CO_2 product. The gas concentration in Figure 3a is defined as

$$Concentration = \frac{x_i}{m_{Fe_2O_3} \times 30\%} \quad (3)$$

where x_i stands for gas mole fraction, $m_{Fe_2O_3}$ is the weight of Fe_2O_3 in the total sample, and 30% is the weight percentage of oxygen in Fe_2O_3, thus the unit is "$\%\cdot g_O^{-1}$".

Small angle X-ray diffraction (SAXRD): Supplementary Figure 3 shows the result of SAXRD patterns of the SBA-15 support, Fe_2O_3@SBA-15 before and after redox cycles. The pore sizes are calculated as 8.3nm and 8.1nm in SBA-15 support and Fe_2O_3@SBA-15, respectively. The

shrinkage of the pore size is consistent with BET measurement, which also suggests that Fe_2O_3@SBA-15 nanochannels are partially filled by Fe_2O_3 nanoparticles. For post redox Fe_2O_3@SBA-15, the pore size is calculated as 7.6nm with a minor extent of decrease, indicating high cyclic stability.

Fixed bed experiment: The conversion of CH_4 and selectivity of syngas were calculated by Supplementary Equations 4 and 5 and plotted in Supplementary Figure 6.

$$\textit{Conversion} = \frac{x_{\mathrm{CH_4,\,in}} - x_{\mathrm{CH_4,\,out}}}{x_{\mathrm{CH_4,\,in}}} \times 100\% \quad (4)$$

$$\textit{Selectivity} = \frac{x_{\mathrm{CO}}}{x_{\mathrm{CO}} + x_{\mathrm{CO_2}}} \times 100\% \quad (5)$$

where $x_{\mathrm{CH_4,\,in}}$ is the CH_4 mole fraction of inlet gas, and $x_{\mathrm{CH_4,\,out}}$ is the CH_4 mole fraction of outlet gas. x_{CO} and $x_{\mathrm{CO_2}}$ stand for mole fraction of CO and CO_2, respectively.

At the four tested WHSV values (17.8, 25, 30, 37.5 mL·$(\mathrm{mg_{Fe2O3}}\ \mathrm{h})^{-1}$), conversion of CH_4 for Fe_2O_3@SBA-15 is 131%, 60%, 76%, 92% higher than unsupported Fe_2O_3, respectively.

Computational details:

The adsorption energy of CH_4 on Fe_2O_3 nanoparticles is calculated using the expression as Supplementary Equation 6:

$$E_{\mathrm{ad}} = (E_{\mathrm{CH_4}} + E_{\mathrm{(Fe_2O_3)}n}) - E_{\mathrm{(CH_4+(Fe_2O_3)}n)} \quad (6)$$

where $E_{\mathrm{CH_4}}$ is the energy of the optimized gas phase geometry of CH_4, $E_{\mathrm{(Fe_2O_3)}n}$ is the total energy of $(Fe_2O_3)_n$ nanoparticle, and $E_{\mathrm{(CH_4+(Fe_2O_3)}n)}$ is the total energy of $(Fe_2O_3)_n$ nanoparticle with adsorbed CH_4. Based on this definition, a more positive E_{ad} corresponds to a more stable configuration. The effect of the temperature is included by explicitly taking into account adsorbed

CH_4 molecules in terms of ab initio atomistic thermodynamics. The change of entropy can be calculated explicitly using Supplementary Equation 7:

$$\Delta S(T) = (\Delta S_{nano}(T) + \Delta S_{CH_4}(T)) - \Delta S_{CH_4@nano}(T) \quad (7)$$

where $\Delta S(T)$ represents the change in entropy as a result of adsorption, $\Delta S_{CH_4@nano}(T)$ represents the entropy of the combined system of a CH_4 molecule adsorbed on the nanoparticle, $S_{nano}(T)$ represents the entropy of the clean nanoparticle, and $S_{CH_4}(T)$ represents the entropy of CH_4 molecule in the gas phase.

Supplementary Figures

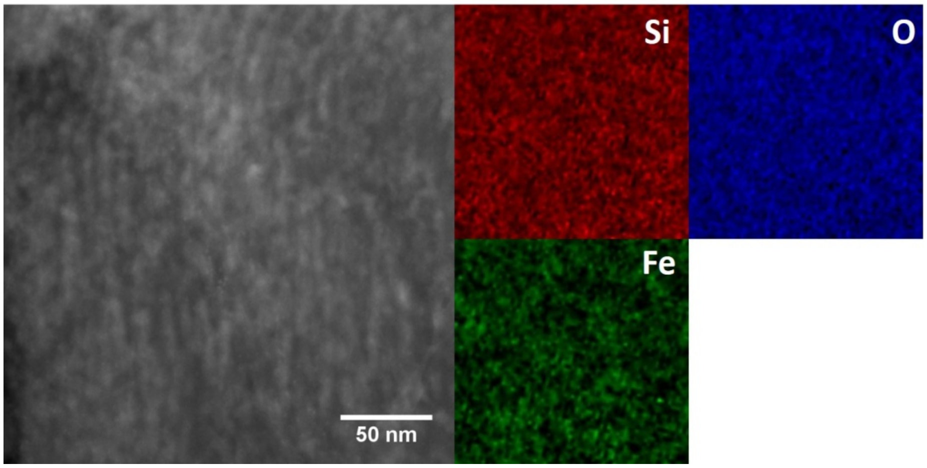

Supplementary Figure 1. EDX mapping of Fe_2O_3@SBA-15

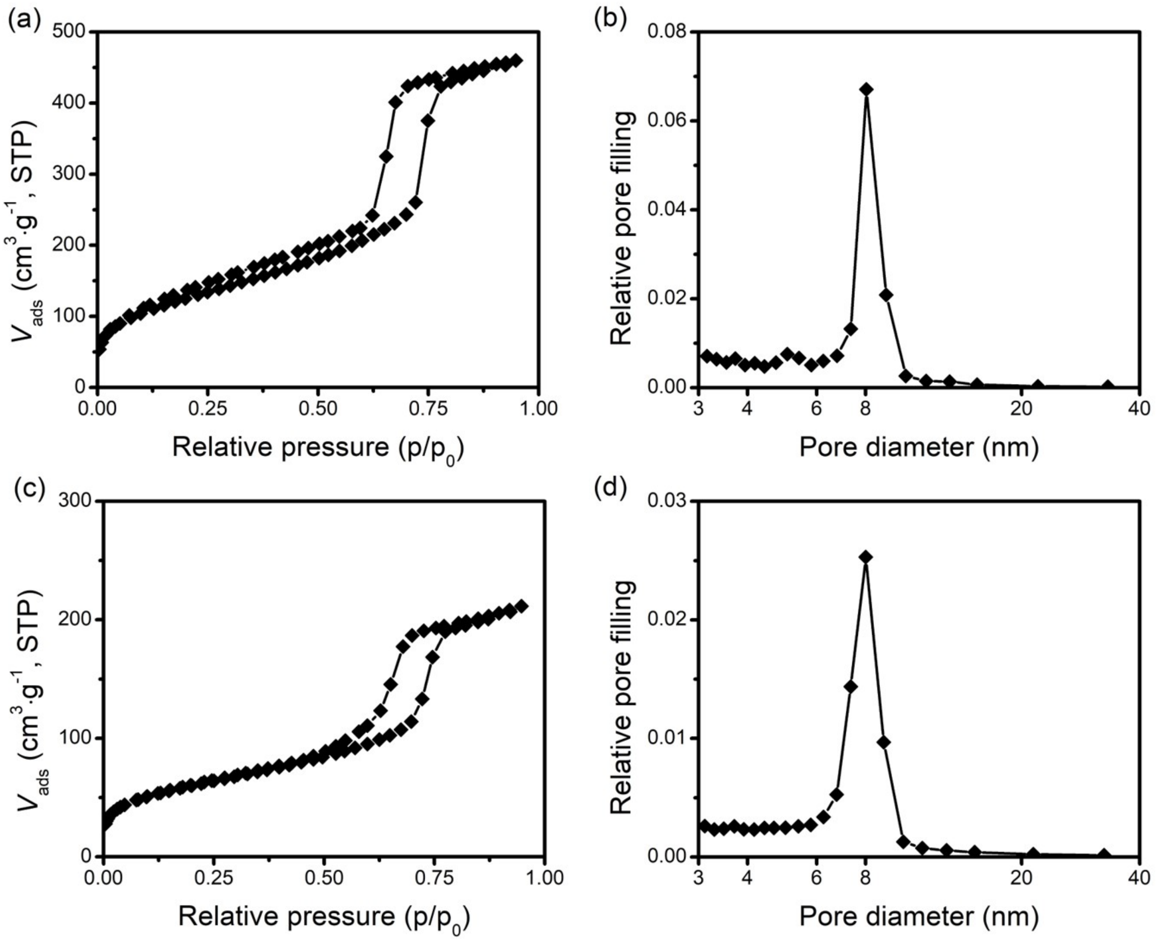

Supplementary Figure 2. Surface and pore size analysis. N_2 sorption isotherms of (a) SBA-15 and (b) its pore size distribution; (c) Fe_2O_3@SBA-15 and (d) its pore size distribution

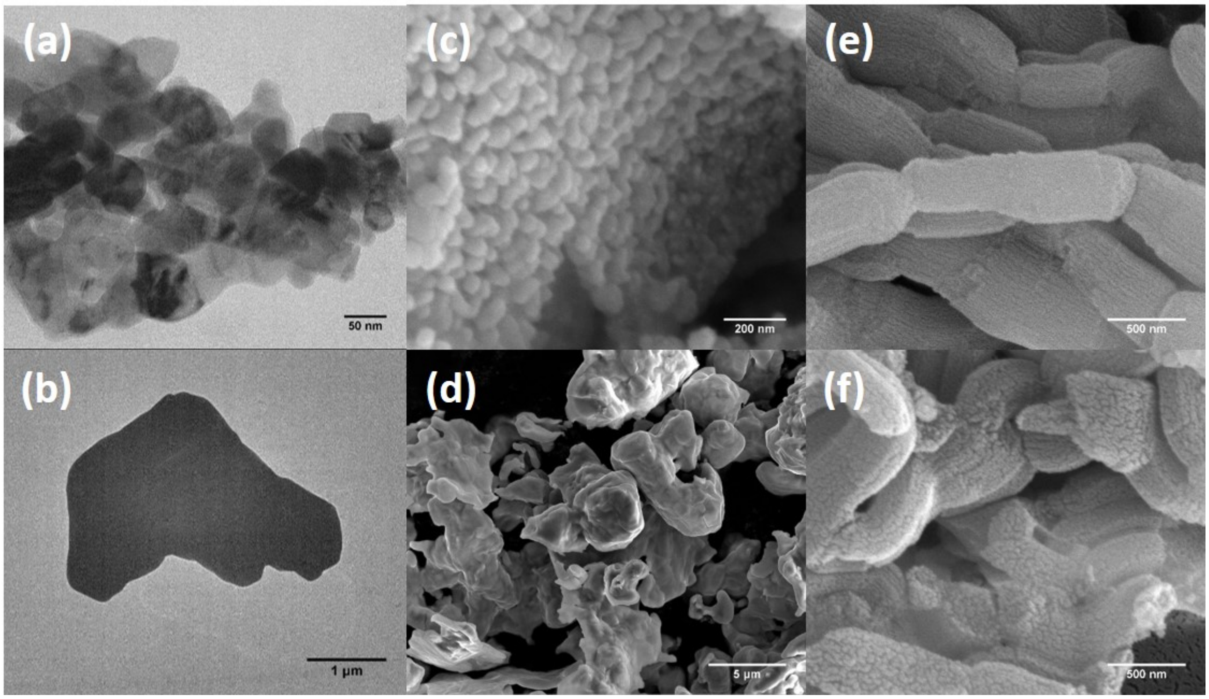

Supplementary Figure 3. Morphology characterization of Fe_2O_3@SBA-15 and unsupported Fe_2O_3 before and after 75 redox cycles. TEM images of (a) fresh unsupported Fe_2O_3; (b) unsupported Fe_2O_3 after 75 redox cycles; SEM images of (c) fresh unsupported Fe_2O_3; (d) unsupported Fe_2O_3 after 75 redox cycles; (e) fresh Fe_2O_3@SBA-15; (f) Fe_2O_3@SBA-15 after 75 redox cycles

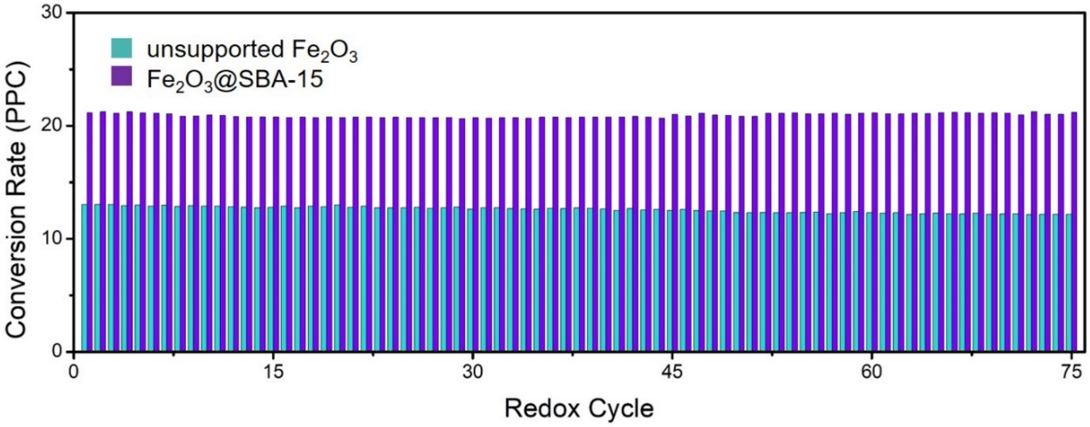

Supplementary Figure 4. Conversion rate in 75 redox cycles for unsupported Fe_2O_3 and Fe_2O_3@SBA-15 at 800^oC

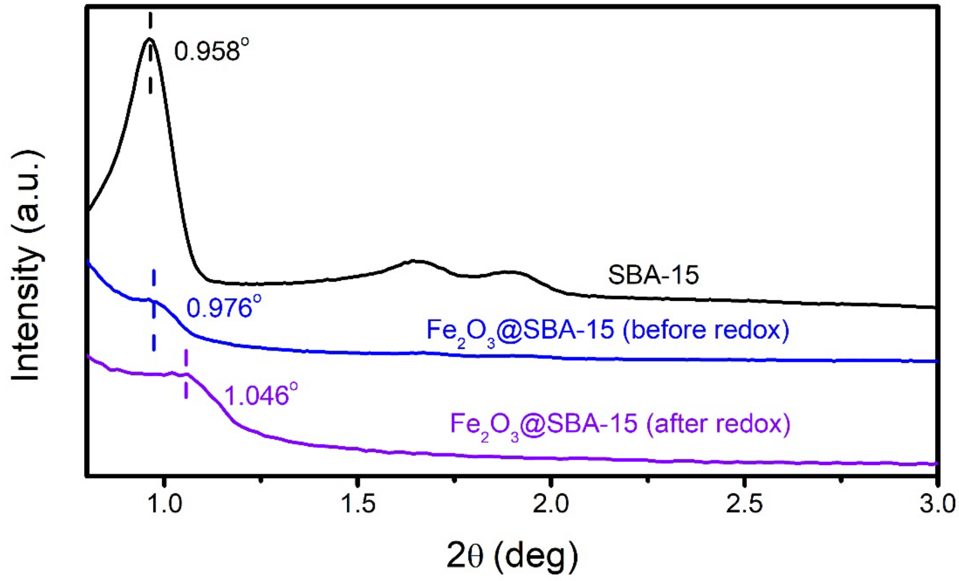

Supplementary Figure 5. SAXRD for SBA-15, Fe_2O_3@SBA-15 before and after 75 redox cycles

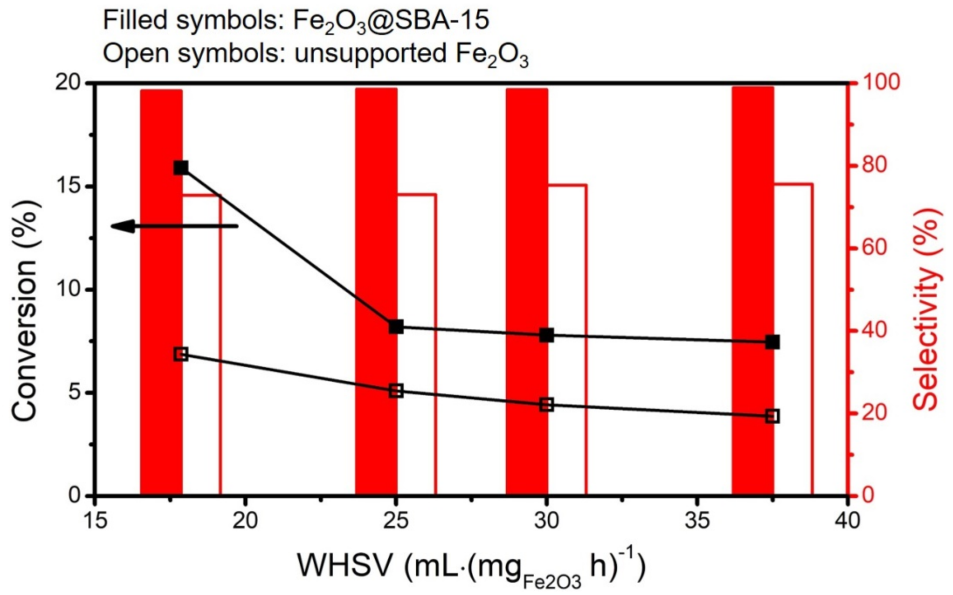

Supplementary Figure 6. Methane conversion and syngas selectivity over unsupported Fe_2O_3 and Fe_2O_3@SBA-15 at different WHSV in the U-tube fixed bed reactor

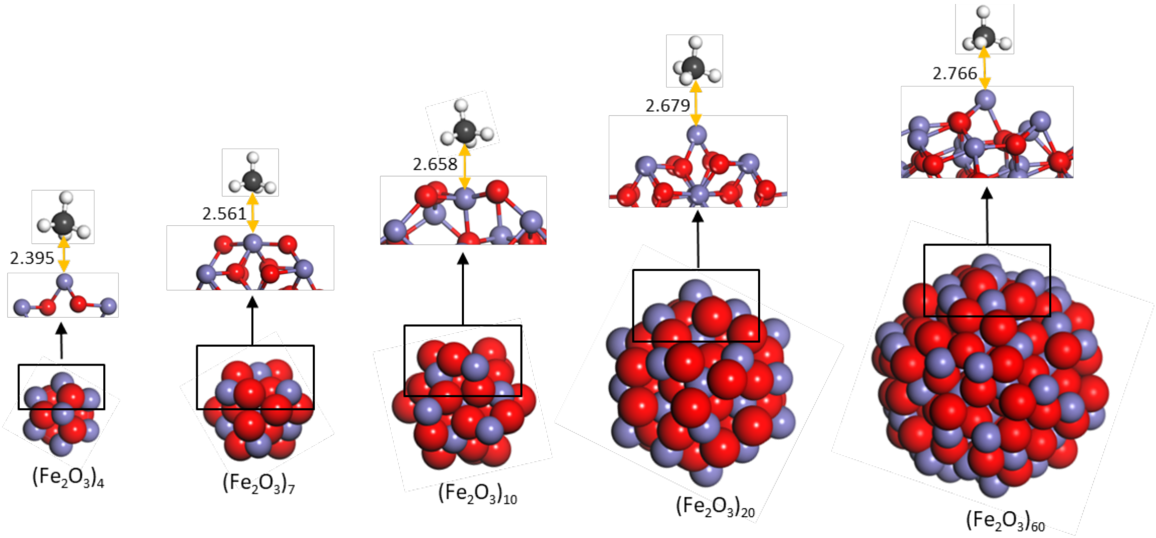

Supplementary Figure 7. Geometrical structures of CH_4 adsorbed on the $(Fe_2O_3)_n$ nanoparticle. The distance between C atom and the nearest Fe atom is indicated.

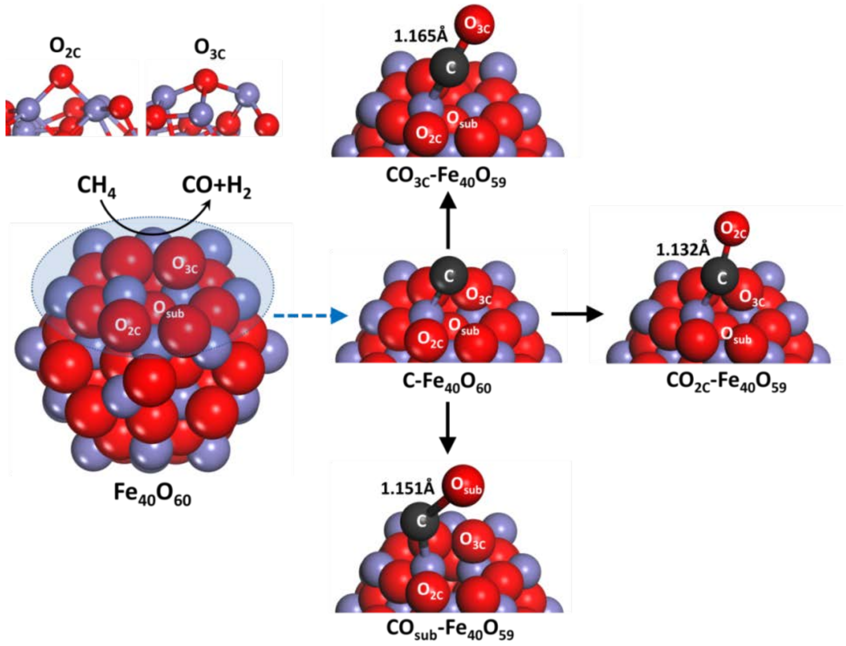

Supplementary Figure 8. The pathway of CO formation on $Fe_{40}O_{60}$ nanoparticle
